# Supplementary material for: Improving provider-initiated testing for HIV and other STI in the primary care setting in Amsterdam, the Netherlands: Results from a multifaceted, educational intervention programme
Source: PLoS One. 2023 Mar 6;18(3):e0282607. doi: 10.1371/journal.pone.0282607 (PMC9987818; doi:10.1371/journal.pone.0282607)
Supplement: S2 Table — Relative test ratios are adjusted for city district of the ordering GP and year of testing. n/a: parameter estimates could not be obtained due to low number. (DOCX) [file pone.0282607.s002.docx]

S2 Table: Adjusted relative overall, urogenital, anorectal and oropharyngeal gonorrhoea test ratios and positivity ratios among GPs in Amsterdam after participation in an educational intervention, compared to GPs before participation, overall and by patient sex and age, 2011-2020.

|  | **Gonorrhoea** **overall** | | **Urogenital gonorrhoea** | | **Anorectal gonorrhoea** | | **Oropharyngeal gonorrhoea** | |
| --- | --- | --- | --- | --- | --- | --- | --- | --- |
|  | **Relative Ratio** | **95% CI** | **Relative Ratio** | **95% CI** | **Relative Ratio** | **95% CI** | **Relative Ratio** | **95% CI** |
| Overall | 0.98 | 0.97 - 0.99 | 0.95 | 0.94 - 0.97 | 1.36 | 1.27 - 1.46 | 1.09 | 1.03 - 1.16 |
| **By sex** | | | | | | | | |
| Males | 1.03 | 1.01 - 1.05 | 0.99 | 0.97 - 1.01 | 1.26 | 1.17 - 1.37 | 1.09 | 1.01 - 1.17 |
| Females | 0.96 | 0.94 - 0.98 | 0.94 | 0.93 - 0.96 | 1.98 | 1.69 - 2.32 | 1.11 | 0.99 - 1.25 |
| **By age categories** | | | | | | | | |
| ≤19 years | 0.92 | 0.86 - 0.98 | 0.90 | 0.84 - 0.96 | 1.10 | 0.63 - 1.92 | 1.04 | 0.66 - 1.66 |
| 20-34 years | 1.00 | 0.98 - 1.01 | 0.98 | 0.96 - 1.00 | 1.48 | 1.30 - 1.67 | 1.08 | 0.98 - 1.20 |
| 34-49 years | 0.91 | 0.88 - 0.93 | 0.88 | 0.86 - 0.90 | 1.28 | 1.15 - 1.43 | 1.02 | 0.92 - 1.13 |
| 50-64 years | 1.12 | 1.07 - 1.16 | 1.06 | 1.01 - 1.11 | 1.43 | 1.23 - 1.65 | 1.27 | 1.11 - 1.47 |
| ≥65 years | 0.83 | 0.74 - 0.93 | 0.80 | 0.71 - 0.91 | 0.87 | 0.58 - 1.30 | 0.93 | 0.63 - 1.39 |
| **Males** **by age categories** | | | | | | | | |
| ≤19 years | 1.14 | 0.99 - 1.32 | 1.10 | 0.95 - 1.27 | 0.90 | 0.37 - 2.19 | n/a | n/a |
| 20-34 years | 1.03 | 0.99 - 1.06 | 1.01 | 0.98 - 1.04 | 1.39 | 1.18 - 1.63 | 1.07 | 0.93 - 1.24 |
| 34-49 years | 0.95 | 0.92 - 0.99 | 0.91 | 0.87 - 0.95 | 1.19 | 1.06 - 1.34 | 1.02 | 0.91 - 1.14 |
| 50-64 years | 1.17 | 1.11 - 1.24 | 1.12 | 1.05 - 1.19 | 1.35 | 1.16 - 1.57 | 1.24 | 1.07 - 1.44 |
| ≥65 years | 0.88 | 0.77 - 1.00 | 0.86 | 0.74 - 1.00 | 0.86 | 0.57 - 1.30 | 0.95 | 0.63 - 1.42 |
| **Females** **by age categories** | | | | | | | | |
| ≤19 years | 0.87 | 0.81 - 0.94 | 0.86 | 0.80 - 0.93 | 1.35 | 0.65 - 2.82 | 0.79 | 0.46 - 1.35 |
| 20-34 years | 1.00 | 0.98 - 1.02 | 0.98 | 0.96 - 1.00 | 1.73 | 1.42 - 2.11 | 1.11 | 0.96 - 1.28 |
| 34-49 years | 0.88 | 0.85 - 0.91 | 0.87 | 0.84 - 0.90 | 2.39 | 1.70 - 3.36 | 1.03 | 0.81 - 1.32 |
| 50-64 years | 1.02 | 0.94 - 1.10 | 0.97 | 0.90 - 1.05 | 3.79 | 2.06 - 6.96 | 1.70 | 1.05 - 2.75 |
| ≥65 years | 0.66 | 0.53 - 0.82 | 0.66 | 0.52 - 0.82 | n/a | n/a | n/a | n/a |
| **Positivity** | | | | | | | | |
| Overall | 1.09 | 1.00 - 1.19 | 1.07 | 0.97 - 1.19 | 0.85 | 0.67 - 1.08 | 1.04 | 0.78 - 1.40 |
| Males | 1.04 | 0.94 - 1.16 | 1.04 | 0.92 - 1.18 | 0.91 | 0.71 - 1.16 | 1.10 | 0.80 - 1.51 |
| Females | 1.07 | 0.90 - 1.27 | 1.05 | 0.88 - 1.26 | 0.82 | 0.25 - 2.69 | 0.90 | 0.40 - 2.01 |

Relative test ratios are adjusted for city district of the ordering GP and year of testing. n/a: parameter estimates could not be obtained due to low numbers.
