# Supplementary material for: Improving provider-initiated testing for HIV and other STI in the primary care setting in Amsterdam, the Netherlands: Results from a multifaceted, educational intervention programme
Source: PLoS One. 2023 Mar 6;18(3):e0282607. doi: 10.1371/journal.pone.0282607 (PMC9987818; doi:10.1371/journal.pone.0282607)
Supplement: S3 Table — *The relative test ratio indicates the number of ordered tests by a GP who participated in the intervention in one quarter, relative to the previous quarter. (DOCX) [file pone.0282607.s003.docx]

S3 Table: Relative trends in HIV, chlamydia and gonorrhoea testing over quarter-year periods among GPs in Amsterdam after participation in an educational intervention, 2011-2020.

| **Test ordered** | **Relative Test Ratio*** | **95% CI** |
| --- | --- | --- |
| HIV | 1.02 | 1.01 - 1.02 |
| **Chlamydia** | | |
| Overall | 1.02 | 1.02 - 1.03 |
| Urogenital | 1.01 | 1.01 - 1.02 |
| Anorectal | 1.09 | 1.08 - 1.10 |
| Oropharyngeal | 1.07 | 1.06 - 1.08 |
| **Gonorrhoea** | | |
| Overall | 1.02 | 1.01 - 1.02 |
| Urogenital | 1.00 | 1.00 - 1.01 |
| Anorectal | 1.09 | 1.08 - 1.10 |
| Oropharyngeal | 1.07 | 1.06 - 1.08 |

*The relative test ratio indicates the number of ordered tests by a GP who participated in the intervention in one quarter, relative to the previous quarter.
